# Fast fixing and comprehensive identification to help improve real-time ligands discovery based on formaldehyde crosslinking, immunoprecipitation and SDS-PAGE separation

**DOI:** 10.1186/1477-5956-12-6

**Published:** 2014-02-01

**Authors:** Lisi Zhu, Menglin Li, Lilong Wei, Xuejiao Liu, Jianrui Yin, Youhe Gao

**Affiliations:** 1 Department of Physiology and Pathophysiology, National Key Laboratory of Medical Molecular Biology, Institute of Basic Medical Sciences, Chinese Academy of Medical Sciences, Peking Union Medical College, Beijing 100005, China; 2 Clinical Laboratory, China-Japan Friendship Hospital, Beijing 100029, China; 3 Department of Nephrology, Beijing An Zhen Hospital of the Capital Medical University, Beijing 100029, China

**Keywords:** Albumin, Formaldehyde cross-linking, Immunoprecipitation, Mass spectrometry, Protein-protein interactions

## Abstract

**Background:**

Fast Fixation is necessary to study real-time protein-protein interactions under physiological conditions. Fast formaldehyde cross-linking can fix transient and weak protein interactions, thereby reducing the number of false negatives but producing great complexity. To reduce this complexity, immunoaffinity purification can Fish out complexes that include particular target proteins, but affinity-based co-purification has a limited capacity to eliminate nonspecific binding to beads and/or antibodies. To Filter out these complexes, SDS-PAGE is used to disrupt non-covalent bonds, thereby eliminating uncross-linked complexes and simultaneously providing molecular weight information for identification.

**Results:**

We described a 4 F strategy to help improve real-time ligands discovery based on formaldehyde crosslinking, immunoprecipitation and SDS-PAGE separation: Fast Fix, Fish, and Filter, using albumin interactome as an example. The use of gel excision without staining makes this strategy comprehensive and sensitive. The target protein must be identified in the same slice as its ligands. The ligands must be identified in slices for the experimental group but not in the corresponding control slices. Only proteins that appear in the range of molecular weights equal to or greater than the sum of the proteins’ theoretical molecular weights, together with the target, are considered ligands. In this study, 5 s of cross-linking with 10% formaldehyde was achieved in human blood. The use of this strategy identified 35 ligands for albumin. Comparison with four major previous studies of the albuminome revealed that 68.57% of the 35 ligands identified in our study were identified in these other studies.

**Conclusions:**

Fast cross-linking was achieved. The 4 F strategy can be used to identify real-time in situ interactions without prior intervention and to comprehensively identify ligands of particular target proteins with fewer false positives.

## Background

Identifying real-time protein-protein interactions is a first step in revealing the mechanisms underlying biological processes. Few proteins function alone, with most functioning in the form of protein complexes. Proteins tend to form various complexes that are constantly associating and disassociating. Transient protein-protein interactions, such as reversible substrate-enzyme binding and receptor-ligand interactions, some of which are weak interactions, are fundamental to many biological processes.

Chemical cross-linking is a useful high-throughput method for studying in situ protein-protein interactions and is able to capture transient and weak interactions. In general, two strategies have been developed in previous cross-linking studies, cross-linking with [1] or without cross-link reversal [2]. In studies without cross-link reversal, the gel is usually stained, and bands of interest are excised and analyzed using LC-MS/MS [2]. Formaldehyde is a powerful zero-length cross-linking reagent that penetrates quickly, inactivates enzymes, and ensures the stability of complexes [3]. The reactions take place rapidly and can be quenched immediately. Formaldehyde has been used for fixation in experiments based on immunohistochemistry, chromatin immunoprecipitation of protein-DNA complexes, mass spectrometry-compatible silver staining, and the examination of protein-protein interactions [3,4]. Paraformaldehyde cross-linking coupled with immunoaffinity chromatography and mass spectrometry has been employed to identify interacting partners of M-Ras, but the shortest incubation time used was 5 min [1]. The amount of cross-linking products generated is determined by the number of protein-protein interactions that exist and the extent of cross-linking. As shown in previous studies, the formaldehyde concentration and incubation time are complementary parameters that can be tuned to achieve efficient cross-linking [5]. **F**ast **F**ixation with a relatively high formaldehyde concentration provides, in effect, a faster shutter speed for capturing images of protein-protein interactions.

Commonly used purification methods, such as co-immunoprecipitation, can **F**ish out target protein complexes. When this strategy is used, stringent washing during immunoprecipitation is unnecessary to remove contaminants, which can be eliminated by comparison with the control. SDS-PAGE is employed to disrupt non-covalent bonds, thereby eliminating uncross-linked complexes and, at the same time, providing molecular weight information as an identification **F**ilter.

**Figure 1:**
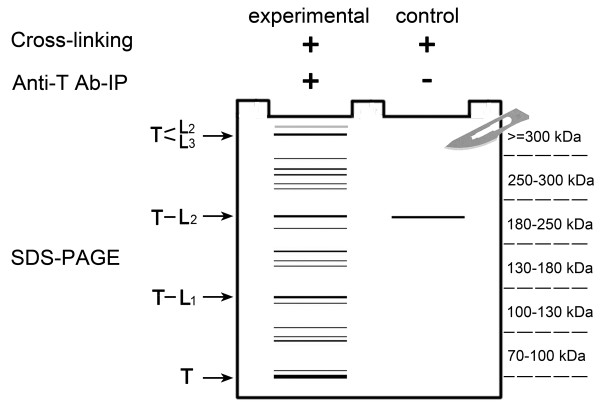
**Principle of the 4 F strategy.** T: target protein, L: ligand. Without staining, the portion of the gel corresponding to molecular weights above that of a single albumin molecule (70 kDa) was excised in six slices using the protein markers as a guide. The slices corresponded to MWs of 70–100 kDa, 100–130 kDa, 130–180 kDa, 180–250 kDa, 250–300 kDa, and greater than 300 kDa. 1. The target protein has to be identified in the same slice as the ligands. 2. The molecular weight of each complex is the sum of the theoretical MWs of the target and the ligand. a) Ligands are not identified in the control, as observed for L_1_ and L_3_. b) Target complexes bind to the beads nonspecifically after cross-linking. To be conservative, the ligand should be identified at a MW higher than that of the control (> = 300 kDa) and outside of the neighboring range (250–300 kDa), as for L2. (L2-T-L3 is an example of a complex with interactions other than binary interactions). 3. Proteins identified in the same range as in the control group or in a neighboring range are eliminated as contaminants and are not shown in the figure.

To obtain true ligands, the two following conditions are required to hold for the proteins in the SDS-PAGE gel after fast cross-linking and immunoprecipitation (Figure 1): 1. the target protein has to be identified in the same slices as the ligands, and 2. the molecular weight of the complexes has to be the sum of the theoretical MWs of the target and the ligand. a) Ligands are not identified in the control, as observed for L_1_ and L_3_. b) Target complexes bind to the beads nonspecifically after cross-linking. To be conservative, the ligand should be identified at a MW higher than that of the control (> = 300 kDa) and outside of the neighboring range (250–300 kDa), as for L_2_. (L2-T-L3 is an example of a complex with interactions other than binary interactions.) Proteins identified in the same range as in the control group or in a neighboring range are eliminated as contaminants and are not shown in the figure.

Albumin was used as an example to illustrate the **4 F** strategy.

## Results and discussion

### Determination of fast fixation conditions

To achieve fast fixation, a 5 s fixation period was tested. Freshly drawn blood was directly added to various concentrations of formaldehyde. Sufficient amounts of 4 M Tris were used to stop the cross-linking reactions. The reaction times were strictly controlled. Samples containing equal volumes of the original blood sample were loaded.

**Figure 2:**
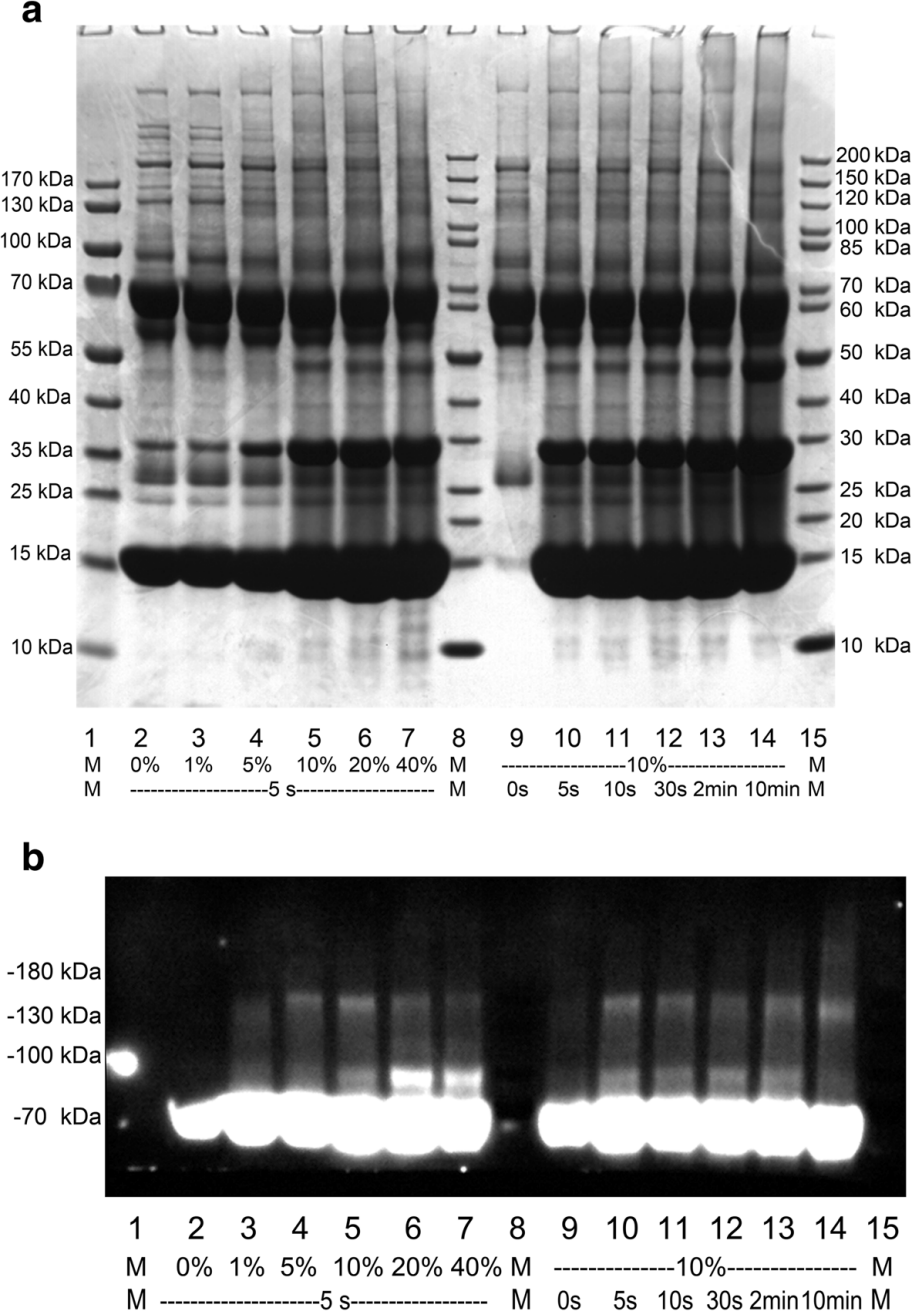
**Formaldehyde cross-linking of human blood samples.** Human blood was incubated with various concentrations of formaldehyde for 5 s or with 10% formaldehyde for various times: **(a)** samples were analyzed using SDS-PAGE and **(b)** samples were analyzed using Western blotting with an anti-albumin antibody.

When using a 5 s duration for the transient cross-linking reaction, the use of formaldehyde at a concentration of 5% or higher for cross-linking resulted in a distinctly different appearance compared with that of the control (Figure 2a). The patterns obtained using 10% and higher concentrations of formaldehyde for cross-linking were similar, indicating that 10% formaldehyde was sufficient for fixing protein-protein interactions in the blood.

To determine whether 5 s was sufficient for cross-linking, longer incubation times with 10% formaldehyde were tested. The patterns obtained with longer incubation times exhibited no obvious differences. There were differences between the patterns for 0 s cross-linking and 0% formaldehyde cross-linking, and the bands at approximately 15 kDa, 25 kDa and 35 kDa might have been caused by hemolysis. The electrophoresis patterns observed for the two sets of cross-linked samples from the two volunteers were nearly the same, indicating good biological and technical reproducibility (Additional file 1: Figure S1).

In the Western blots produced using an albumin antibody (Figure 2b), two bands and smears at molecular weights higher than that of albumin were observed in all of the cross-linked samples but not in the controls. Higher concentrations and longer cross-linking times did not result in a significant increase in the number of cross-linking products obtained. Therefore, a 5 s cross-linking time in 10% formaldehyde was chosen for the following experiments. However, most of the albumin molecules existed in an uncross-linked state.

### Identification of albumin ligands

To identify the interacting partners of the target protein, an excess of antibody was used for immunoprecipitation to purify sufficient amounts of cross-linked products for MS analysis. After immunoprecipitation, a 4-12% denaturing gradient gel was used to achieve good separation of protein complexes with high molecular weights. Without staining, the portion of the gel corresponding to molecular weights greater than 70 kDa was excised and sectioned into six slices using the molecular weight markers as a guide. Samples from the experimental group and control group were analyzed using LC-MS/MS. Different LC columns were used for the samples and controls to reduce cross-contamination.

After the Mascot search, the significance threshold and ion score cut-off were set to 0.05 using MudPIT protein scoring. The original peptide sequences for identified proteins in each molecular weight range can be referenced in additional file (Additional file 2: Table S1). Only proteins that had at least two significant sequences were reported using the report builder. Proteins in the contaminants database or that contained “keratin” or “keratinocyte” in their description were excluded. There were 55 proteins in the experimental groups, and 33 were identified in the control samples (Additional file 3: Table S2).

To identify true ligands, the following two conditions are required (as illustrated in Figure 1): 1. the target protein must be identified in the same slice as the ligands, and 2. the molecular weight of a complex must be the sum of the theoretical MWs of the target and the ligand. a) Ligands are not identified in the control, as observed for L_1_ and L_3_. b) Target complexes bind to the beads nonspecifically after cross-linking. To be conservative, the ligand should be identified at a MW higher than that of the control (> = 300 kDa) and outside the neighboring range (250–300 kDa), as for L_2_. (L2-T-L3 is an example of complexes with interactions other than binary interactions). Proteins identified in the same range as in the control group or in a neighboring range are eliminated as contaminants and are not shown in the figure. A total of 35 proteins were considered to be albumin ligands (Table 1), 27 of which were not identified in the control. Peptide match data for above identified albumin ligands were listed in Table S2 (Additional file 4: Table S3).

**Table 1:** Spectrum counts for albumin and the identified albumin ligands in each molecular weight range

**ID**	**Accession**	**Previously reported**	**Theoretical MW (kDa)**	**Observed MW range (kDa)**					
				**≥300**	**250-300**	**180-250**	**130-180**	**100-130**	**70-100**
P02768	ALBU_HUMAN^a^		71.317	59 (45)	184 (68)	269 (109)	418 (74)	328 (70)	516 (103)
P02760	AMBP_HUMAN^a^	14	39.886		8	2		0 (3)	
P01024	CO3_HUMAN^a^	12, 13, 14	188.569		55	49	43 (31)		3
P15924	DESP_HUMAN^a^	13	334.021	3				0 (2)	
P02671	FIBA_HUMAN^a^	11, 12, 13, 14	95.656	8	23	10	6 (5)	8	5 (15)
P69905	HBA_HUMAN^a^	13	15.305	3	9	10	9	19 (4)	16 (8)
P01871	IGHM_HUMAN^a^	12, 14	49.96	2	8	9	3	3	4 (29)
P12273	PIP_HUMAN^a^		16.847	3	7		0 (2)		
P00747	PLMN_HUMAN^a^	12, 13, 14	93.247			2	3	30 (3)	
P01023	A2MG_HUMAN	12, 14	164.613	13	53	47			
P02647	APOA1_HUMAN	11, 12, 13, 14	30.759					3	2
P05090	APOD_HUMAN	12, 14	21.547		7				
P02730	B3AT_HUMAN		102.013	3	4	4	2	19	5
P04040	CATA_HUMAN		59.947				2		
P00450	CERU_HUMAN	12, 13, 14	122.983				2		
P00751	CFAB_HUMAN	11, 12, 13, 14	86.847					5	
P16452	EPB42_HUMAN		77.816	4					
Q01469	FABP5_HUMAN		15.497		7				
P02675	FIBB_HUMAN	12, 14	56.577	7	30	23	21	15	2
P02679	FIBG_HUMAN	12	52.106		23	10	7	8	
P05155	IC1_HUMAN	11, 13, 14	55.347					2	
P01857	IGHG1_HUMAN	11, 12, 14	36.596		8	11	27	31	20
P01859	IGHG2_HUMAN	11, 12, 14	36.505		3	6	9		
P01860	IGHG3_HUMAN	12, 14	42.287			8	19	22	14
P01861	IGHG4_HUMAN	14	36.431					19	
P01834	IGKC_HUMAN	11, 12, 14	11.773		2				5
B9A064	IGLL5_HUMAN		23.391			3			
P06312	KV401_HUMAN		13.486		17				
P0CG04	LAC1_HUMAN	12, 14	11.512					4	7
P47929	LEG7_HUMAN		15.123		2				
P32119	PRDX2_HUMAN		22.049					4	4
P31151	S10A7_HUMAN		11.578		8				
P06702	S10A9_HUMAN	14	13.291		10				
P29508	SPB3_HUMAN		44.594		9				
P04004	VTNC_HUMAN	12, 13, 14	55.069				3		
P25311	ZA2G_HUMAN	13	34.465		4				
Total spectrum counts in each MW range				40	288	184	147	173	71

^a^Albumin and ligands also identified in the control.

The spectrum counts for the control are listed in brackets.

The comparison of the 35 albumin ligands with the 1926 plasma proteins listed in order of descending abundance based on peptide-spectrum matches (Farrah T et al.) [6] indicated that the cross-linking products were not produced by random cross-linking with highly abundant proteins in the blood. Only 5 of the top 20 and 15 of the top 100 plasma proteins were identified as albumin ligands in our results. The four most abundant proteins, which include albumin, transferrin, alpha-1-antitrypsin, and retinol-binding protein 4, were not identified as ligands. In addition, the abundances of ten ligands identified in our study are so low that these proteins are not even present in the human plasma proteome reference set (Farrah T et al.) [6].

Albumin has many ligands and is a member of many complexes. Thus, albumin was present in every slice in the experimental group in this study. Other target proteins with fewer ligands may be present in only narrow molecular weight ranges.

When this strategy is used, stringent washing during immunoprecipitation is unnecessary to exclude contaminants because they can be eliminated by comparison with the control. Because albumin was immunoprecipitated using gentle washing and because albumin is the most abundant protein in the blood, it was also present in the control slices. Other target proteins may not appear in the control slices as often.

Irrelevant cross-linked complexes exhibiting nonspecific binding to beads and proteins with post-translational modifications might appear in slices corresponding to higher molecular weights than those of potential complexes. A control consisting of a cross-linked sample without an antibody was used to eliminate both possibilities, which cannot be achieved using an uncross-linked control. To be conservative, the proteins in the experimental samples that appeared in the corresponding slice as in the control samples or in a neighboring slice were not considered true ligands. For example, for complement C3 (P01024, CO3_HUMAN), 31 spectra were identified in the molecular range of 130–180 kDa in the control, and the 43 and 49 spectra identified in the corresponding range of 130–180 kDa and the neighboring range of 180–250 kDa were not sufficient to warrant accepting complement C3 (P01024, CO3_HUMAN) as a true ligand. In total, 55 spectra identified in the 250–300 kDa slice qualified as true ligands in this study, as for L_2_ in Figure 1.

Two obvious bands observed in the Western blots fell in the observed molecular weight ranges of 70–100 kDa and 130–180 kDa, and these bands could not be explained by the identified ligands. These bands might have been due to the presence of dimeric or modified albumin.

AMBP, alpha-1-microglobulin/bikunin precursor, has been reported to interact with albumin [7]. Albumin-AMBP complexes were isolated using anti-AMBP affinity chromatography and were separated under native conditions. The complexes were a mixture of 1:1 and 1:2 complexes, with masses determined by SDS/PAGE of approximately 100 kDa and 135 kDa, respectively [7]. The plasma concentration of albumin-AMBP was estimated to be 5.2 mg/L [7]. If the molecular weight criteria had not been applied, the AMBP protein would have been regarded as a contaminant because it also appeared in the control group.

AMBP has also been reported to form a complex with A2M [8]. The total theoretical molecular weight of the ALB-AMBP-A2M complex is 270 kDa. In this experiment, both AMBP and A2M were identified in the range of 250–300 kDa, indicating that the complex was cross-linked and captured by the anti-albumin antibody.

### Comparison with previous studies addressing albumin-binding proteins

Albumin binds proteins and peptides [9,10]. Four major studies have been conducted on the human albuminome, all of which were based on affinity purification using an anti-albumin antibody, an anti-albumin column or an albumin column directly. Totals of 125 (Zhou, M. et al.) [11], 50 (Gundry, R. et al.) [12], 62 (Scumaci, D. et al.) [13] and 151 (Holewinski, R. et al.) [14] ligands were identified in these studies. The comparison of our results with those of these four albuminome studies revealed that 68.57% of the 35 ligands identified in our study were also identified in these previous studies (Table 1). Eleven ligands were identified only by our strategy.

Low-molecular-weight proteins/peptides that associate with albumin and five other high-abundance proteins in the serum have been studied using affinity columns [11]. However, the use of different elution conditions resulted in poor overlap. Twenty-eight ligands were identified using antibody immunoprecipitation, and 107 ligands were identified using an affinity column in the same study. Only 10 of these ligands were identified by both methods.

## Conclusions

This study represents the first use of transient cross-linking to identify protein-protein interactions. The use of fast fixation and molecular weight filtering in this new method reduces the number of random results and makes the results comprehensive and precise. In addition, gel excision without staining makes this **4 F** strategy sensitive. Stringent washing during immunoprecipitation is unnecessary for excluding contaminants because they can be eliminated by comparison with the control.

When this strategy is used, only proteins that simultaneously satisfy the following three criteria can be considered false positives: 1. the putative ligand binds to the antibody by forming a complex with other proteins; 2. the protein is present in ranges with molecular weights equal to or greater than the sum of the theoretical molecular weights of the potential ligands plus the target protein in SDS-PAGE; and 3. the protein is present in the same range as the target protein.

False negatives can occur only when formaldehyde cannot access the protein interaction interface within the allotted cross-linking time.

The use of this strategy identified 35 ligands for albumin. Comparison with the results of three previous major studies of the albuminome revealed that 68.57% of the 35 ligands identified in our study were identified in previous albuminome studies.

There was a concern that the action of adding blood samples to formaldehyde might result in unnatural protein contacts and produce random cross-linking. In this case, higher-abundance proteins should have been more likely to bind to albumin. However, the four most abundant proteins [6], which include albumin, transferrin, alpha-1-antitrypsin, and retinol-binding protein 4, were not identified as ligands. This potential problem may not affect the results when **4 F** strategy is used to analyze cells or tissues.

This **4 F** strategy can be used to identify real-time in situ interactions without prior intervention and to comprehensively identify ligands of particular target proteins with fewer false positives. Therefore, this method can potentially change how protein-protein interaction research is performed.

## Methods

### Ethics statement

The two healthy volunteers had provided written informed consent to participate in the study and publish these case details. The Institutional Review Board (IRB) of Institute of Basic Medical Sciences, Chinese Academy of Medical Sciences had approved this consent procedure. The project number is 019–2013.

The IRB had approved the blood draw procedure and had reviewed the proposed use of human subjects in the above-mentioned project. The right and the welfare of the subjects are adequately protected; the potential risks are outweighed by potential benefits.

### Antibodies and reagents

Formaldehyde was purchased from Sinopharm Chemical Reagent Co., Beijing. Ultrapure Tris was purchased from the USB Corporation (Affymetrix, Santa Clara, California, USA). All of the antibodies used were purchased from commercial sources. These antibodies included a mouse monoclonal anti-human serum albumin antibody for immunocytochemistry applications (ab10241, Abcam, Boston, MA, USA) and a peroxidase-conjugated AffiniPure goat anti-mouse IgG (H + L) (ZB-2305, ZSGB-bio, Beijing, China). Protein A + G agarose for co-immunoprecipitation was purchased from the Beyotime Institute of Biotechnology (P2012, Shanghai, China). PBS for washing was obtained from the Cell Culture Center at Peking Union Medical College (Beijing, China).

For protein electrophoresis, 1.5 mm × 10 well 4-12% Bis-Tris gels (NuPAGE, Life Technologies, Carlsbad, California, USA) were used. The 5× SDS loading buffer was obtained from Genestar (Shanghai, China), and the 1851 protein marker was obtained from Fermentas (Waltham, Massachusetts, USA). For trypsin digestion, trypsin gold of mass spectrometry grade was purchased from Promega (Madison, WI, USA). IAA (iodoacetamide) was purchased from GE (Fairfield, Connecticut, USA), and DTT (dithiothreitol) was purchased from Merck (Whitehouse Station, New Jersey, USA). All other regents were of high purity.

### Fast formaldehyde cross-linking

Solutions of 40% formaldehyde were diluted to 1%, 5%, 10%, and 20% using double-distilled water. The final mass concentration of NaCl was 0.9% in the diluted solutions. Several 50 mL centrifuge tubes containing 4 mL of these formaldehyde solutions were prepared. For the quenching of the different concentrations of formaldehyde, 4 M Tris, pre-prepared in 50 mL tubes, was used. A volume of 2 mL was used for formaldehyde concentrations of 10% and below, 4 mL was used for 20% formaldehyde, and 8 mL was used for 40% formaldehyde. Freshly drawn blood samples (5 mL) were collected from two healthy male volunteers into tubes without anticoagulants and divided carefully into several 1.5 mL Eppendorf tubes. Aliquots of 0.5 mL of blood were added to the 50 mL tubes containing formaldehyde. After cross-linking, 4 M Tris was added, and the contents of the tubes were mixed gently. The cross-linking times were strictly controlled. Mixing formaldehyde and Tris before adding to blood is the control of 0 s cross-linking. After cross-linking and quenching, the samples were centrifuged at 300 g for 10 min at 4°C, and the supernatants were then dispensed into small aliquots and stored at -80°C. All of the samples used were thawed only once. Hemolysis occurred after quenching with Tris but was ignored because it had no impact in this study.

### Immunoprecipitation and SDS-PAGE separation

To capture all of the cross-linked and uncross-linked albumin, an excess of antibody was used for immunoprecipitation. Two 70 μL cross-linking samples from one volunteer were diluted with 500 μL of PBS and precleared twice with 150 μL of beads each time and were then incubated with 125 μL (250 μg) of an anti-human serum albumin antibody or PBS for 16 hours. Then, 200 μL of Protein A + G agarose beads was added, followed by incubation for 3 hours. The agarose beads were washed five times using PBS (1 mL per wash). After the last wash, the supernatant was discarded, and the agarose beads were resuspended in 320 μL of 2.5 × SDS loading buffer (160 μL for the control group) at 4°C before use. The samples were heated for 10 min at 65°C before electrophoresis [1]. Aliquots of 20 μL of the supernatants were loaded into the lanes of 4-12% SDS-PAGE gels.

### LC-MS/MS

Without staining, the portion of each gel corresponding to molecular weights above that of a single albumin molecule (70 kDa) was excised in six slices using the protein markers as a MW guide. The slices corresponded to MWs of 70–100 kDa, 100–130 kDa, 130–180 kDa, 180–250 kDa, 250–300 kDa, and greater than 300 kDa. All of the slices were subjected to overnight in-gel trypsinization at 37°C. Peptides were extracted using 5% formic acid/50% acetonitrile followed by 2.5% formic acid/50% acetonitrile, both for 1 hour at 37°C. The extracts were mixed together and dried under vacuum. Visible solids were re-dissolved using 0.1% formic acid and desalted using a Waters Oasis® HLB 1 cc Extraction Cartridge (Waters Corporation, Milford, Massachusetts, USA). The elution fractions obtained in 90% acetonitrile were dried again and stored at -80°C until use.

The samples were dissolved in 10 μL of 0.1% formic acid prior to LC-MS/MS analysis. The samples were loaded using a maximum volume of 8 μL and were separated using an Agilent 1200 HPLC system at a flow rate of 500 μL/min on in-house-produced 100 μm × 150 mm C18 capillary columns packed with Sunchrom packing material (SP-120-3-ODS-AQ, 3 μm, The Great Eur-Asia Sci-Tech Development Co. Ltd, Beijing, China). The samples were eluted using water/ACN/formic acid gradients over 120 min and analyzed using LTQ Orbitrap Velos mass spectrometry (Thermo Fisher Scientific). The experimental group and the control group were analyzed separately using two different columns. MS scans were acquired over the 300–2000 m/z range with the resolution set to a value of 60,000, and the 20 most intense ions in each full MS scan were selected for CID MS/MS analysis. Dynamic exclusion was set to 15 s.

The raw MS files were processed using Distiller 2.4.3 and searched using MASCOT 2.4.0. First, an error-tolerant search was employed to discover the most frequent modifications because formaldehyde has been reported to modify amino acids [5]. The maximum mass deviations for the parent and fragment ions were set to 5 ppm and 0.5 Da, respectively. The MS/MS data were analyzed and matched to protein sequences in the SwissProt_2012_07 database, with the taxonomy filter, and the contaminants_20120713 database. The most frequent modifications—acetylation (N-term), loss of ammonia (N-term C), carbamidomethylation (DEHY), carbamidomethylation (N-term), deamidation (NQ), formyl (N-term) and oxidation (M)—were specified as variable modifications. The fixed modification was carbamidomethylation (C). Mascot searches were conducted with a maximum of two missed cleavages, a peptide mass tolerance of 5 ppm, and a fragment mass tolerance of 0.8 Da.

## Competing interests

The authors declare that they have no competing interests.

## Authors’ contributions

YG proposed the project. LZ designed the experiments. LZ and JY collected samples. LZ and ML performed the experiments. LW and XL performed mass spectrometry analysis. LZ performed the data analysis. LZ and YG wrote the manuscript. All authors read and approved the final manuscript.

## Supplementary Material

Additional file 1: Figure S1 Formaldehyde cross-linking of human blood samples from two volunteers. Human blood was incubated with various concentrations of formaldehyde for 5 s or with 10% formaldehyde for various times, samples were analyzed using SDS-PAGE.The electrophoresis patterns observed in two sets of cross-linked samples from two volunteers (figure S1A and figure S1B were patterns of samples from volunteer A and B, respectively) were nearly the same, which indicated good biological and technical reproducibility. Click here for file

Additional file 2: Table S1 Original peptide sequences for identified proteins in each molecular weight range. Click here for file

Additional file 3: Table S2 Number of Significant Matches in Albumin Immunoprecipitation. Click here for file

Additional file 4: Table S3 Peptide match data for identified proteins in each molecular weight range. Click here for file
