# Supplementary material for: Fast fixing and comprehensive identification to help improve real-time ligands discovery based on formaldehyde crosslinking, immunoprecipitation and SDS-PAGE separation
Source: Proteome Sci. 2014 Feb 1;12:6. doi: 10.1186/1477-5956-12-6 (PMC3922604; doi:10.1186/1477-5956-12-6)

Figure S1 Formaldehyde cross-linking of human blood samples from two volunteers.

Human blood was incubated with various concentrations of formaldehyde for 5 s or with 10% formaldehyde for various times, samples were analyzed using SDS-PAGE. The electrophoresis patterns observed in two sets of cross-linked samples from two volunteers (figure S1A and figure S1B were patterns of samples from volunteer A and B, respectively) were nearly the same, which indicated good biological and technical reproducibility.

A B


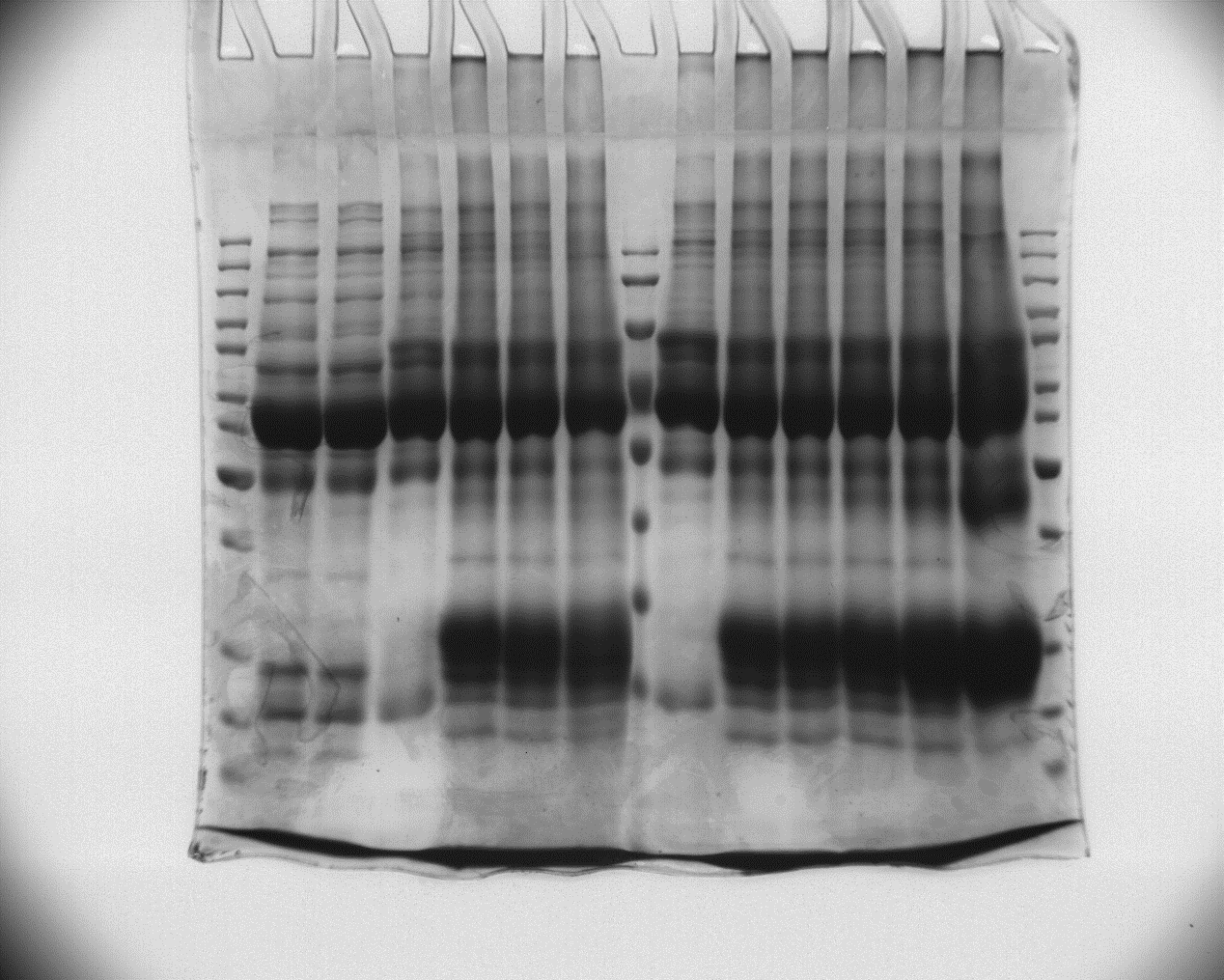

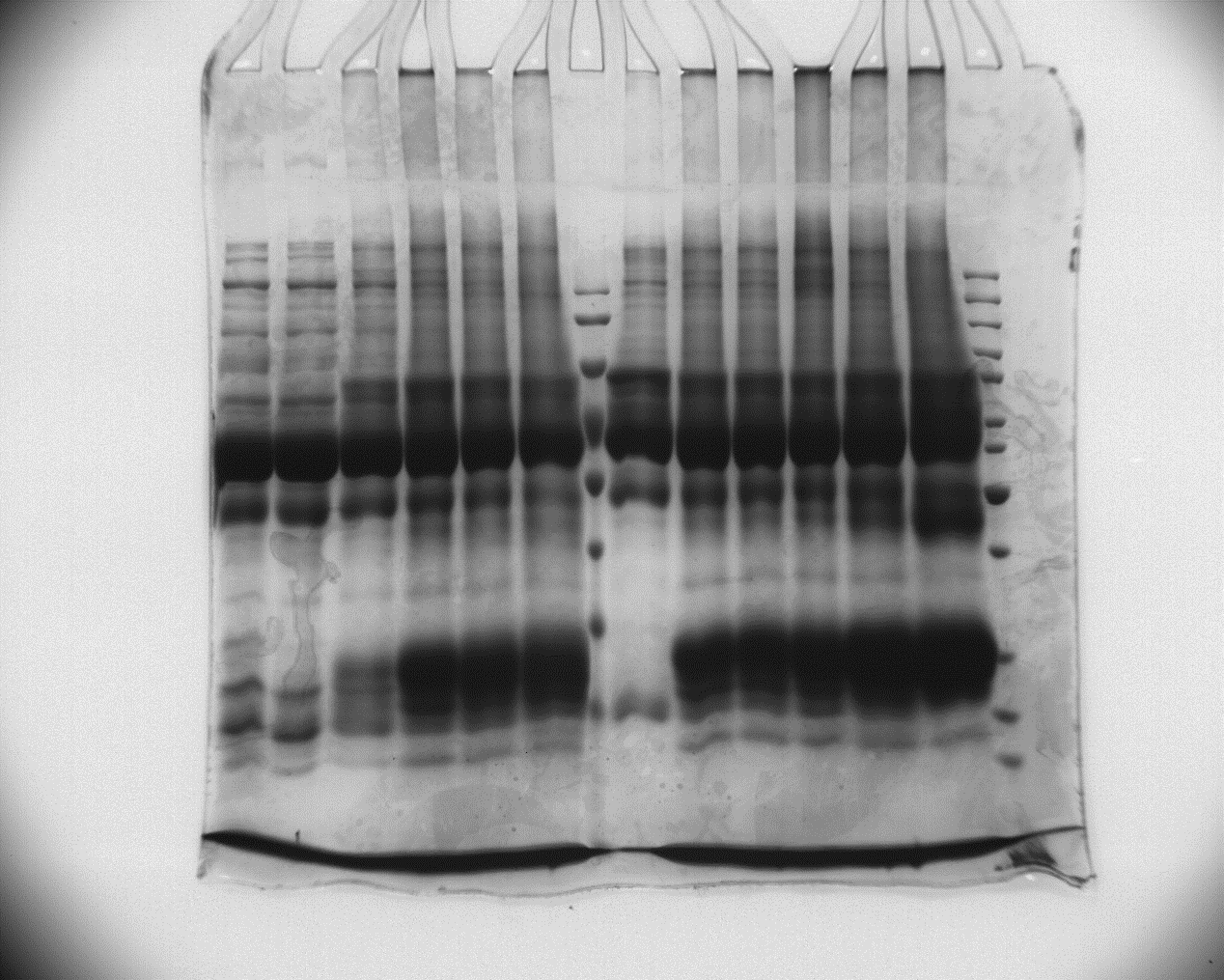

Supplement: Additional file 1: Figure S1 — Formaldehyde cross-linking of human blood samples from two volunteers. Human blood was incubated with various concentrations of formaldehyde for 5 s or with 10% formaldehyde for various times, samples were analyzed using SDS-PAGE.The electrophoresis patterns observed in two sets of cross-linked samples from two volunteers (figure S1A and figure S1B were patterns of samples from volunteer A and B, respectively) were nearly the same, which indicated good biological and technical reproducibility. [file 1477-5956-12-6-S1.doc]
