# Supplementary material for: Fast fixing and comprehensive identification to help improve real-time ligands discovery based on formaldehyde crosslinking, immunoprecipitation and SDS-PAGE separation
Source: Proteome Sci. 2014 Feb 1;12:6. doi: 10.1186/1477-5956-12-6 (PMC3922604; doi:10.1186/1477-5956-12-6)
Supplement: Additional file 3: Table S2 — Number of Significant Matches in Albumin Immunoprecipitation. [file 1477-5956-12-6-S3.doc]

Table S2 Number of Significant Matches in Albumin Immunoprecipitation

| ALB-Ab (The experimental group) | | | | | | | | | ALB-PBS (The control group) | | | | | | | | |
| --- | --- | --- | --- | --- | --- | --- | --- | --- | --- | --- | --- | --- | --- | --- | --- | --- | --- |
| GN | Accession | Mass | >300 | 250-300 | 180-250 | 130-180 | 100-130 | 70-100 | GN | Accession | Mass | >300 | 250-300 | 180-250 | 130-180 | 100-130 | 70-100 |
|  |  |  | 230-300 | 180-230 | 110-180 | 60-110 | 30-60 | 0-30 |  |  |  | 230-300 | 180-230 | 110-180 | 60-110 | 30-60 | 0-30 |
| ACTB | ACTB_HUMAN | 42.052 |  | 2 |  |  |  |  | ACTB | ACTB_HUMAN | 42.052 |  |  | 10 |  |  |  |
| ALB | ALBU_HUMAN | 71.317 | 59 | 184 | 269 | 418 | 328 | 516 | ALB | ALBU_HUMAN | 71.317 | 45 | 68 | 109 | 74 | 70 | 103 |
| AMBP | AMBP_HUMAN | 39.886 |  | 8 | 2 |  |  |  | AMBP | AMBP_HUMAN | 39.886 |  |  |  |  | 3 |  |
| ANK1 | ANK1_HUMAN | 207.334 | 3 | 8 |  |  |  |  | ANK1 | ANK1_HUMAN | 207.334 |  | 6 |  |  |  |  |
| APOB | APOB_HUMAN | 516.651 | 9 |  |  |  |  |  | APOB | APOB_HUMAN | 516.651 | 2 |  |  |  |  |  |
| CFH | CFAH_HUMAN | 143.68 |  | 7 | 13 |  |  |  | CFH | CFAH_HUMAN | 143.68 |  |  | 6 |  |  |  |
| C3 | CO3_HUMAN | 188.569 |  | 55 | 49 | 43 |  | 3 | C3 | CO3_HUMAN | 188.569 |  |  |  | 31 |  |  |
| DCD | DCD_HUMAN | 11.391 | 15 |  |  | 8 | 9 | 13 | DCD | DCD_HUMAN | 11.391 | 12 | 10 | 7 | 11 | 8 | 8 |
| DEFA1 | DEF1_HUMAN | 10.536 |  | 6 |  |  |  |  | DEFA1 | DEF1_HUMAN | 10.536 |  |  | 6 |  |  |  |
| DSP | DESP_HUMAN | 334.021 | 3 |  |  |  |  |  | DSP | DESP_HUMAN | 334.021 |  |  |  |  | 2 |  |
| DSG1 | DSG1_HUMAN | 114.702 | 3 |  |  |  | 2 |  | DSG1 | DSG1_HUMAN | 114.702 | 3 |  |  |  |  |  |
| FGA | FIBA_HUMAN | 95.656 | 8 | 23 | 10 | 6 | 8 | 5 | FGA | FIBA_HUMAN | 95.656 |  |  |  | 5 |  | 15 |
| FLG2 | FILA2_HUMAN | 249.296 | 6 | 3 | 2 |  | 4 | 2 | FLG2 | FILA2_HUMAN | 249.296 | 6 | 2 |  | 4 | 6 |  |
| FN1 | FINC_HUMAN | 266.052 | 5 | 7 |  |  |  |  | FN1 | FINC_HUMAN | 266.052 |  | 17 |  |  |  |  |
| HBA1 | HBA_HUMAN | 15.305 | 3 | 9 | 10 | 9 | 19 | 16 | HBA1 | HBA_HUMAN | 15.305 |  |  |  |  | 4 | 8 |
| HBB | HBB_HUMAN | 16.102 | 13 | 24 | 23 | 21 | 34 | 43 | HBB | HBB_HUMAN | 16.102 | 4 | 6 | 5 | 5 | 17 | 11 |
| HRNR | HORN_HUMAN | 283.14 | 13 | 6 | 2 | 4 | 10 |  | HRNR | HORN_HUMAN | 283.14 | 6 |  | 2 | 2 | 5 | 5 |
| IGHM | IGHM_HUMAN | 49.96 | 2 | 8 | 9 | 3 | 3 | 4 | IGHM | IGHM_HUMAN | 49.96 |  |  |  |  |  | 29 |
| ITIH1 | ITIH1_HUMAN | 101.782 |  | 12 |  |  |  |  | ITIH1 | ITIH1_HUMAN | 101.782 |  | 2 |  |  |  |  |
| ITIH2 | ITIH2_HUMAN | 106.853 |  | 16 | 4 |  |  |  | ITIH2 | ITIH2_HUMAN | 106.853 |  | 8 |  |  | 7 | 6 |
| ITIH4 | ITIH4_HUMAN | 103.521 |  |  |  | 6 |  |  | ITIH4 | ITIH4_HUMAN | 103.521 |  |  |  | 5 |  |  |
| PIP | PIP_HUMAN | 16.847 | 3 | 7 |  |  |  |  | PIP | PIP_HUMAN | 16.847 |  |  |  | 2 |  |  |
| PLG | PLMN_HUMAN | 93.247 |  |  | 2 | 3 | 30 |  | PLG | PLMN_HUMAN | 93.247 |  |  |  |  | 3 |  |
| S100A8 | S10A8_HUMAN | 10.885 |  | 14 |  |  |  |  | S100A8 | S10A8_HUMAN | 10.885 |  |  | 7 |  |  |  |
| SPRR2D | SPR2D_HUMAN | 8.584 |  | 32 |  |  | 5 |  | SPRR2D | SPR2D_HUMAN | 8.584 | 4 |  | 11 | 7 | 2 |  |
| SPTA1 | SPTA1_HUMAN | 281.039 | 7 |  |  |  |  |  | SPTA1 | SPTA1_HUMAN | 281.039 | 7 | 28 | 3 |  |  |  |
| SPTB | SPTB1_HUMAN | 247.171 | 5 |  |  |  |  |  | SPTB | SPTB1_HUMAN | 247.171 |  | 15 |  |  |  |  |
| A2M | A2MG_HUMAN | 164.613 | 13 | 53 | 47 |  |  |  | ANXA1 | ANXA1_HUMAN | 38.918 |  |  | 11 |  |  |  |
| APOA1 | APOA1_HUMAN | 30.759 |  |  |  |  | 3 | 2 | ANXA2 | ANXA2_HUMAN | 38.808 |  |  | 3 |  |  |  |
| APOD | APOD_HUMAN | 21.547 |  | 7 |  |  |  |  | BPIFB1 | BPIB1_HUMAN | 52.58 |  | 4 |  |  |  |  |
| SLC4A1 | B3AT_HUMAN | 102.013 | 3 | 4 | 4 | 2 | 19 | 5 | CRNN | CRNN_HUMAN | 53.73 |  |  | 11 |  |  |  |
| CAT | CATA_HUMAN | 59.947 |  |  |  | 2 |  |  | SPRR3 | SPRR3_HUMAN | 18.598 |  |  | 6 |  |  |  |
| CP | CERU_HUMAN | 122.983 |  |  |  | 2 |  |  | TGM3 | TGM3_HUMAN | 76.926 |  |  | 3 |  |  |  |
| CFB | CFAB_HUMAN | 86.847 |  |  |  |  | 5 |  | PRSS1 | TRY1_HUMAN | 27.111 | 20 |  |  |  | 26 |  |
| EPB42 | EPB42_HUMAN | 77.816 | 4 |  |  |  |  |  | ZG16B | ZG16B_HUMAN | 22.725 |  |  | 3 |  |  |  |
| FABP5 | FABP5_HUMAN | 15.497 |  | 7 |  |  |  |  |  |  |  |  |  |  |  |  |  |
| FGB | FIBB_HUMAN | 56.577 | 7 | 30 | 23 | 21 | 15 | 2 |  |  |  |  |  |  |  |  |  |
| FGG | FIBG_HUMAN | 52.106 |  | 23 | 10 | 7 | 8 |  |  |  |  |  |  |  |  |  |  |
| SERPING1 | IC1_HUMAN | 55.347 |  |  |  |  | 2 |  |  |  |  |  |  |  |  |  |  |
| IGHG1 | IGHG1_HUMAN | 36.596 |  | 8 | 11 | 27 | 31 | 20 |  |  |  |  |  |  |  |  |  |
| IGHG2 | IGHG2_HUMAN | 36.505 |  | 3 | 6 | 9 |  |  |  |  |  |  |  |  |  |  |  |
| IGHG3 | IGHG3_HUMAN | 42.287 |  |  | 8 | 19 | 22 | 14 |  |  |  |  |  |  |  |  |  |
| IGHG4 | IGHG4_HUMAN | 36.431 |  |  |  |  | 19 |  |  |  |  |  |  |  |  |  |  |
| IGKC | IGKC_HUMAN | 11.773 |  | 2 |  |  |  | 5 |  |  |  |  |  |  |  |  |  |
| IGLL5 | IGLL5_HUMAN | 23.391 |  |  | 3 |  |  |  |  |  |  |  |  |  |  |  |  |
| IGKV4-1 | KV401_HUMAN | 13.486 |  | 17 |  |  |  |  |  |  |  |  |  |  |  |  |  |
| IGLC1 | LAC1_HUMAN | 11.512 |  |  |  |  | 4 | 7 |  |  |  |  |  |  |  |  |  |
| LGALS7 | LEG7_HUMAN | 15.123 |  | 2 |  |  |  |  |  |  |  |  |  |  |  |  |  |
| PRDX2 | PRDX2_HUMAN | 22.049 |  |  |  |  | 4 | 4 |  |  |  |  |  |  |  |  |  |
| S100A7 | S10A7_HUMAN | 11.578 |  | 8 |  |  |  |  |  |  |  |  |  |  |  |  |  |
| S100A9 | S10A9_HUMAN | 13.291 |  | 10 |  |  |  |  |  |  |  |  |  |  |  |  |  |
| SERPINB3 | SPB3_HUMAN | 44.594 |  | 9 |  |  |  |  |  |  |  |  |  |  |  |  |  |
| SPRR1B | SPR1B_HUMAN | 10.337 |  | 2 |  |  |  |  |  |  |  |  |  |  |  |  |  |
| SPRR2F | SPR2F_HUMAN | 8.541 |  | 28 |  |  |  |  |  |  |  |  |  |  |  |  |  |
| TF | TRFE_HUMAN | 79.294 |  |  |  |  |  | 11 |  |  |  |  |  |  |  |  |  |
| VTN | VTNC_HUMAN | 55.069 |  |  |  | 3 |  |  |  |  |  |  |  |  |  |  |  |
| AZGP1 | ZA2G_HUMAN | 34.465 |  | 4 |  |  |  |  |  |  |  |  |  |  |  |  |  |
